# Residual Energy Analysis in Cognitive Radios with Energy Harvesting UAV under Reliability and Secrecy Constraints

**DOI:** 10.3390/s20102998

**Published:** 2020-05-25

**Authors:** Waqas Khalid, Heejung Yu, Song Noh

**Affiliations:** 1 Department of Electronics and Information Engineering, Korea University, Sejong 30019, Korea; waqas283@gmail.com; 2 Department of Information and Telecommunication Engineering, Incheon National University, Incheon 22012, Korea

**Keywords:** unmanned aerial vehicle, cognitive radio, spectrum sensing, energy harvesting, connection outage probability, secrecy outage probability

## Abstract

The integration of unmanned aerial vehicles (UAVs) with a cognitive radio (CR) technology can improve the spectrum utilization. However, UAV network services demand reliable and secure communications, along with energy efficiency to prolong battery life. We consider an energy harvesting UAV (e.g., surveillance drone) flying periodically in a circular track around a ground-mounted primary transmitter. The UAV, with limited-energy budget, harvests radio frequency energy and uses the primary spectrum band opportunistically. To obtain intuitive insight into the performance of energy-harvesting, and reliable and secure communications, the closed-form expressions of the residual energy, connection outage probability, and secrecy outage probability, respectively, are analytically derived. We construct the optimization problems of residual energy with reliable and secure communications, under scenarios without and with an eavesdropper, respectively, and the analytical solutions are obtained with the approximation of perfect sensing. The numerical simulations verify the analytical results and identify the requirements of length of sensing phase and transmit power for the maximum residual energy in both reliable and secure communication scenarios. Additionally, it is shown that the residual energy in secure communication is lower than that in reliable communication.

## 1. Introduction

Traditional wireless spectrum standards rely on the static spectrum allocation policies where a specific frequency band is assigned to the specific licensed users. Such a policy causes unbalanced spectrum utilization and degrades the spectral efficiency. Therefore, strict spectrum allocation is insufficient to meet the ever-growing demands of spectral resources for futuristic networks such as the internet of things (IoT), and 5G [1,2]. The flexible spectrum allocation and efficient spectrum utilization can be achieved with the cognitive radio (CR) technologies. A dynamic spectrum access (DSA) approach enables the time-division multiple access based spectrum sharing among CR users and mitigates the spectrum scarcity and under-utilization issues [3,4,5]. Thus, CR is the most promising paradigm for wireless communications, and is one of the potential technologies adopted for futuristic networks. The DSA-based approach is classified into underlay, overlay, and interweave/opportunistic spectrum access (OSA). In underlay approach, secondary users (SUs), i.e., CR users and primary users (PUs), transmit simultaneously. However, the SUs have to transmit with lower power to guarantee low interference to the PUs. The concurrent transmissions are possible in overlay approach also, with the SUs assisting the primary transmissions using any relaying techniques. However, the SUs can exploit only the spectral opportunities in interweave/OSA based approach, i.e., the SUs transmit only when the spectral holes are detected. Thus, the spectral efficiency of OSA-CR networks highly depends on the successful integration of the PUs and SUs, and requires the SUs to be capable of performing the reliable sensing or keeping precise track of the primary transmissions. Hence, the reliable spectrum sensing is one of the crucial components in OSA-CR networks. Spectrum sensing can be performed either individually or cooperatively. The perfect spectrum sensing is impossible to achieve in realistic wireless fading scenarios. Hence, the sensing errors due to imperfect sensing should be considered for the precise analyses of CR network performance. The sensing performance in imperfect sensing scenarios can be enhanced by choosing the optimal sensing operating point, i.e., a pair of false alarm and detection probabilities [6,7].

Moreover, signals in open and shared wireless medium are vulnerable to the eavesdropping, i.e., data interception by the illegitimate eavesdroppers. Traditionally, secure wireless data transmission requires cryptographic techniques at network layer. However, the information-security based on encryption and cryptographic techniques is not sufficient for the secure communications because advances in hardware design significantly increase the computational capabilities of the eavesdroppers. Different from the traditional cryptography algorithms, physical layer security (PLS) utilizes the transmission techniques and inherent properties of the wireless medium. The PLS is considered one of the potential solutions for the secure communication in wireless channels [8,9]. The PLS controls the physical signal, i.e., makes it decodable for only the legitimate user. In the PLS, the difference between the channel capacities of the main data link, i.e., between the source and destination, and the wiretap link, i.e., between the source and eavesdropper, is termed as the secrecy capacity, which is a performance measure for the PLS. Additionally, the probabilities of secrecy outage for a given secrecy rate is used to evaluate the secrecy performance [10,11].

On the other hand, unmanned aerial vehicles (UAVs) have been used for various applications, such as monitoring, surveying, data transmission/communication, aerial remote sensing, product delivery, traffic control, and agriculture mapping [12]. However, UAVs typically operate within the unlicensed spectrum bands (e.g., IEEE-S, IEEE-L, ISM), thus competing with a large number of other devices in the popular wireless networks, including WiFi, WiMAX, and Bluetooth. It is unavoidable for the UAVs to face the spectrum scarcity in the near future. The CR technology overcomes the spectrum overcrowd and scarcity issues for the UAVs. The use of UAVs in CR networks also improves the sensing performance because of the short distance line-of-sight (LoS) communication and the higher strength signal without ground fading and shadowing. However, unlike the other ground transceivers with external power supplies, the UAVs are limited by their on-board battery power. Consequently, most of the UAV applications are designed for the short communication periods where permanent access to a costly spectrum-band is not required. The UAV-infrastructures can provide only the temporary services to the areas of interest unless the issue of battery-life is addressed [13].

Furthermore, energy harvesting (EH) in wireless networks is the process of extracting energy from the surrounding environment, such as from solar, heat, wind, and radio frequency (RF) signals [14,15]. The EH techniques have come to the forefront to improve the battery’s limited capacity, i.e., supply energy to the energy-constrained nodes. The ambient RF-EH is a safe and convenient source of energy since it carries energy and information simultaneously. The simultaneous information and power transfer becomes possible through the power splitting devices, and the radio signals are converted into the usable DC power. The RF-EH is used in the wireless networks due to wide availability of radio sources, e.g., WiFi networks, radio broadcasting towers, base stations, and mobile phones. With the recent advances, the RF-EH is also utilized on the CR networks as a greenery solution. Thus, for the above mentioned situation, the transmission from one node (e.g., PU) can provide power to any other node (e.g., SU) which is receiving or listening to the transmission [16,17].

### 1.1. Contributions

The main contributions of this paper are summarized as follows:

- We consider the energy management aspect of the energy harvesting CR-based UAV with limited-energy budget. The closed-form expressions of the total residual energy, connection outage probability, and secrecy outage probability are derived under a circular flight condition.
- We aim to extend the on-board battery life-time for UAV by maximizing the energy obtained through the EH and minimizing the transmission energy consumption. Thus, the optimal lengths of sensing phase and the transmit powers are obtained by solving the formulated optimization problems of maximum residual energy under the constraints of connection and secrecy outage probabilities with perfect sensing approximation.
- The analytical results are verified through the numerical simulations including imperfect sensing. Based on the results, we provide guidelines in designing an energy harvesting UAV-based CR system with the reliable and secure communications under scenarios without and with an eavesdropper, respectively.

### 1.2. Paper Organization

The rest of the paper is organized as follows. In Section 2, we briefly describe the system model. The sensing procedure and signal model are explained in Section 3. The residual energy, and the connection and secrecy outage probabilities are investigated in Section 4 and Section 5, respectively. Section 6 presents the optimization of total residual energy under scenarios without and with an eavesdropper. Section 7 provides numerical simulations and discussion. Finally, conclusions are presented in Section 8.

## 2. System Model

**Figure 1:**
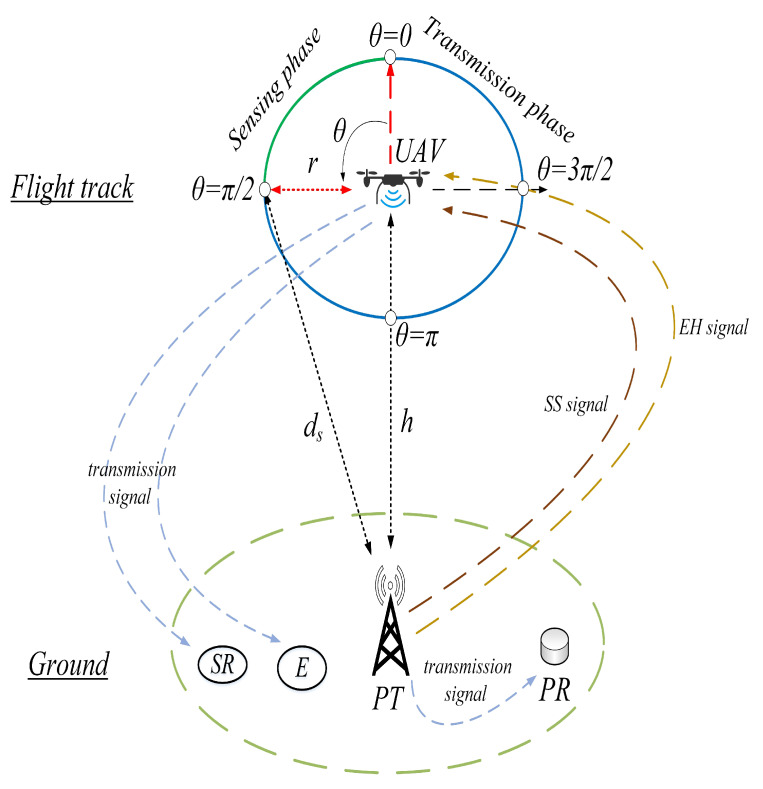
System model.

The system model consists of an energy-constrained UAV (e.g., surveillance drone) as a secondary (cognitive) transmitter, a corresponding secondary receiver (SR), a ground-mounted primary transmitter (PT) and receiver (PR) pair, and an eavesdropper (E), as shown in Figure 1. An energy harvesting UAV (EH-UAV) flies in a circular fight track with a constant velocity (speed) *v* with PT locating at the center. The altitude of EH-UAV relative to the PT is *h*, and the radius of circular track is given by *r*. The on-board power supply is responsible for the energy required for the flight operations, i.e., hovering and transition, with recharging possible only after the completion of each flight. The EH-UAV opportunistically exploits the primary-band, i.e., owned by PT and PR, in the absence or presence of E. The PT and PR communicate probabilistically during the flight of EH-UAV, which is divided into the sensing and transmission radians, i.e., sensing and transmission periods. In the sensing phase with a duration of *t*, the EH-UAV simultaneously harvests RF energy from the received signal and performs the spectrum sensing (SS) procedure for the opportunistic use of the primary band. The dynamic power splitting device splits the received primary signal into the two power fractions of (1−ρ) and ρ for EH and SS purposes, respectively, where ρ denotes the power splitting factor [15]. In the transmission phase with a duration of $T_{d}$, the EH-UAV performs the full-proportioned EH, i.e., ρ=0, if a signal from PT is detected. Otherwise, the EH-UAV sends its data to SR with the portion of powers gathered in the sensing phase through EH unit, and on-board supply. We consider the linear EH model, i.e., the harvested power is linearly proportional to the received RF power [16,17]. During the circular flight, the EH-UAV consumes energy in SS and EH procedures during the sensing phase, and in EH or information-transmission procedures during the transmission phase, respectively. In cellular systems, the sector antennas are generally used for the ground-stations, with projection of the radiation pattern determines the coverage area. Moreover, a down-tilt setting strategy is considered when the base station is located in higher altitude than the user equipment [18]. In proposed scenario, the ground-mounted PT is assumed to be equipped with the omnidirectional antenna. The reason is that only the received signal-to-noise ratio (SNR) at the UAV, irrespective of the signal characteristic, is considered. For example, a base station with three sectors transmits the three different signals via the three sector antennas. However, in terms of the signal energy (not the individual signal), it can be regarded as the omnidirectional pattern. The sector antennas for a PT, i.e., a base station, with a down-tilt setting strategy can also be considered. However, the received sensing SNR at the UAV will be lowered for such a scenario. Moreover, because the primary signal is considered only for the energy detection, the detail model for the primary signal is not required. The signal transmission from PT across the channel is modeled by the two-state Markov chain model, with random variables representing the duration of each state following the exponential distribution. Moreover, $P_{on}$ and $P_{off}$ are the stationary probabilities for the ON and OFF states, respectively [19,20]. An ON state indicates that the channel is being used by the PT (unavailable for the EH-UAV), while the OFF state indicates that the channel is free and there is no activity of PT.

## 3. Sensing Procedure and Signal Modeling

The simplest non-coherent energy detection method is considered for the spectrum sensing at the EH-UAV because it has low computational complexity and does not involve any complicated signal processing. The target signal, i.e., the signal from PT, is detected by comparing the measured signal energy with a pre-determined sensing threshold. It does not need any prior knowledge of the target signal [21]. In the sensing phase, the *n*th samples of the signals received for the SS and EH, denoted by $y_{ss}\left(n\right)$ and $y_{eh}\left(n\right)$, are expressed as

(1) $$\begin{array}{cc} y_{ss}\left(n\right) & =\sqrt{\rho }h_{p}x\left(n\right)+\sqrt{\rho }w\left(n\right), \end{array}$$

(2) $$\begin{array}{cc} y_{eh}\left(n\right) & =\sqrt{\left(1-\rho \right)}h_{p}x\left(n\right)+\sqrt{\left(1-\rho \right)}w\left(n\right). \end{array}$$

The decision metric, ξ, representing the received signal power, for the sensing procedure is given as

(3) $$\xi =\frac{1}{N}\sum_{n=1}^{N}{\left|y_{ss}\left(n\right)\right|}^{2}$$

where x(n) is the transmit signal from the PT, which is assumed to be a complex-valued phase shift keying (PSK) with power $P_{x}=E\left[{\left|x\left(n\right)\right|}^{2}\right]$ and *N* is the total number of samples in the sensing phase. Let $h_{p}$ be the channel fading coefficient of the link between the PT and EH-UAV. The instantaneous power gain of the channel, $|h_{p}{|}^{2}$, is an exponentially distributed random variable, i.e., $Exp(\lambda_{p})$, where $\lambda_{p}$ is the rate parameter [19,20,21]. The mean of $|h_{p}{|}^{2}$ is given by $\omega_{p}=1/\lambda_{p}=d_{p}^{\kappa }$, where $d_{p}$ denotes the distance between the respective nodes, and κ denotes the path loss exponent. The channel gains of all links are assumed to be quasi-static fading, i.e., the channel gains remain constant during the entire circular flight period, i.e., 2π. Moreover, w(n) is the noise at the EH-UAV, which is modeled by a circularly symmetric complex Gaussian (CSCG) distribution, i.e., $w\left(n\right)∼\mathrm{CN}\left(0,\sigma^{2}\right)$.

By using the central limit theorem, for large *N*, the decision statistic ξ can be approximated with the Gaussian distribution with mean $\rho \sigma^{2}$ and variance $\frac{\rho^{2}\sigma^{4}}{N}$, i.e., $\xi ∼N\left(\rho \sigma^{2},\frac{\rho^{2}\sigma^{4}}{N}\right)$, under null hypothesis $H_{0}$. Similarly, under the alternative hypothesis $H_{1}$, the decision statistic is given by $N\left(\left(1+\gamma \right)\rho \sigma^{2},\frac{\left(1+2\gamma \right)\rho^{2}\sigma^{4}}{N}\right)$. Here, $H_{0}$ describes a situation in which a signal from PT does not exist, and $H_{1}$ expresses the case in which a signal from PT exists. We consider that the $\sigma^{2}$ is accurately known at the UAV; thus, a proper sensing threshold, ϵ, design is possible. Substituting the above statistical properties under each hypothesis, the probabilities of detection ($P_{d}$) and false alarm ($P_{fa}$) are expressed as [22]

(4) $$\begin{array}{cc} P_{d} & =Q\left(\left(\frac{\epsilon }{\rho \sigma^{2}}-\gamma -1\right)\sqrt{\frac{r\theta f_{s}}{v(2\gamma +1)}}\right), \end{array}$$

(5) $$\begin{array}{cc} P_{fa} & =Q\left(\left(\frac{\epsilon }{\rho \sigma^{2}}-1\right)\sqrt{\frac{r\theta f_{s}}{v}}\right). \end{array}$$

where θ∈(0,2π) denotes the sensing radian and 2π−θ is referred to the transmission radian. Additionally, $\gamma =\frac{|h_{p}{|}^{2}P_{x}}{\sigma^{2}}$ is the signal-to-noise ratio (SNR) of the sensing signal at the EH-UAV, and $f_{s}$ is the sampling frequency. Q(.) is complement of the standard normal distribution function, often denoted as $Q\left(x\right)=\int_{x}^{\infty }\frac{1}{\sqrt{2\pi }}e^{-\frac{-t^{2}}{2}}dt$, and is simply referred to as the Q-function. This represents the tail probability of the standard Gaussian distribution [21].

Based on Equations (4) and (5), the $P_{fa}$ with the target $P_{d}$, i.e., $P_{fa}$ as a function of $P_{d}$, is expressed as [22]

(6) $$P_{fa}=Q\left(\sqrt{2\gamma +1}Q^{-}\left(P_{d}\right)+\frac{r\theta f_{s}}{v}\gamma \right).$$

## 4. Residual Energy Analysis

The sensing and transmission durations, i.e., *t* and $T_{d}$, respectively, are given by

(7) $$\begin{array}{cc} t & =\frac{r\theta }{v}, \end{array}$$

(8) $$\begin{array}{cc} T_{d} & =\frac{r(2\pi -\theta )}{v}. \end{array}$$

Moreover, the spectrum sensing distance $d_{s}$ is given by,

(9) $$\begin{array}{c} d_{s}=\sqrt{r^{2}+h^{2}} \end{array}$$

Given Equations (7) and (8), we can investigate the residual energy for both durations.

### 4.1. Sensing Phase

In the sensing phase, the harvested energy by the EH-UAV is given as

(10) $$E_{H,s}=\frac{r}{v}\theta \left(1-\rho \right)\eta P_{p},$$

where η is the energy conversion efficiency of the EH circuit. $P_{p}$ denotes the average power of the received signal, which is defined as $P_{p}=P_{on}\left(\right|h_{p}{|}^{2}P_{x}+\sigma^{2})+P_{off}\sigma^{2}$. In general, the EH unit cannot harvest energy when the channel is idle, i.e., when only the noise is received. However, interference from other transmitters, e.g., from another cell, can be regarded as noise. In such a case, the EH unit can harvest energy even though the amount of harvested energy is negligible. Therefore, we consider both the cases where the primary signal is in ON and OFF states. The EH-UAV requires energy to operate the EH circuit, and it is independent of the instantaneous received signal power. The energy consumed for operating the EH unit is given as

(11) $$E_{C1,s}=\frac{r}{v}\theta \left(1-\rho \right)P_{w},$$

where $P_{w}$ is the fixed power consumption to run the EH unit. For spectrum sensing, the EH-UAV also consumes a fixed power $P_{\delta }$ [23]. Hence, the energy consumption in spectrum sensing is given as

(12) $$E_{C2,s}=\frac{r}{v}\theta P_{\delta },$$

From Equations (10)–(12), the residual energy, i.e., difference between the harvested and consumed energies, is given as

(13) $$E_{R,s}=E_{H,s}-E_{C1,s}-E_{C2,s}\Rightarrow \frac{r}{v}\theta \left\{\left\{\left(1-\rho \right)\left(\eta P_{p}-P_{w}\right)\right\}-P_{\delta }\right\}.$$

### 4.2. Transmission Phase

In the transmission phase, the harvested energy can be obtained as

(14) $$E_{H,t}=\frac{r}{v}\left(2\pi -\theta \right)\eta P_{t},$$

where $P_{t}$ is the average power of the received signal, which is defined as $P_{t}=P_{on}P_{d}\left(\right|h_{p}{|}^{2}P_{x}+\sigma^{2})+P_{off}P_{fa}\sigma^{2}$. The energy consumed during the IT procedure is expressed as

(15) $$E_{C1,t}=\frac{r}{v}\left(2\pi -\theta \right)P_{it},$$

where $P_{it}$ is the average power consumption for the secondary data transmission, which is defined as $P_{it}=P_{on}\left(1-P_{d}\right)P_{tx}+P_{off}\left(1-P_{fa}\right)P_{tx}$. Here, $P_{tx}$ is the power of the transmit signal. Moreover, the energy consumed for operating the EH unit is given as

(16) $$E_{C2,t}=\frac{r}{v}\left(2\pi -\theta \right)P_{w}P_{bc},$$

where $P_{w}$ is the fixed power consumption for EH operation and $P_{bc}=P_{d}P_{on}+P_{fa}P_{off}$ is the probability that the EH operation is working when the EH-UAV senses the channel to be busy, i.e., detection and false alarm events.

From Equations (14)–(16), the residual energy in the transmission phase is given as,

(17) $$\begin{array}{cc} E_{R,t} & =E_{H,t}-E_{C1,t}-E_{C2,t}\\ \; & =\frac{r}{v}\left(2\pi -\theta \right)\left\{\eta P_{t}-P_{it}-P_{w}P_{bc}\right\}. \end{array}$$

### 4.3. Total Residual Energy

With Equations (13) and (17), the total residual energy after a single flight period (2π) of the EH-UAV can be written as

(18) $$\begin{array}{cc} E_{R,Tot} & =E_{R,s}+E_{R,t}\\ \; & =\frac{r}{v}\theta \left\{\left\{\left(1-\rho \right)\left(\eta P_{p}-P_{w}\right)\right\}-P_{\delta }\right\}+\frac{r}{v}\left(2\pi -\theta \right)\left\{\eta P_{t}-P_{it}-P_{w}P_{bc}\right\}\\ \; & =\frac{r}{v}\theta \left\{\left\{\left(1-\rho \right)\left(\eta \left(P_{on}\left(\right|h_{p}{|}^{2}P_{x}+\sigma^{2})+P_{off}\sigma^{2}\right)-P_{w}\right)\right\}-P_{\delta }\right\}\\ \; & \;\;\;\;\;+\frac{r}{v}\left(2\pi -\theta \right)\left\{\eta \left(P_{on}P_{d}\left(\right|h_{p}{|}^{2}P_{x}+\sigma^{2})+P_{off}P_{fa}\sigma^{2}\right)\;-\;\left(P_{on}\left(1-P_{d}\right)P_{tx}+P_{off}\left(1\;-\;P_{fa}\right)P_{tx}\right)\right.\\ \; & \;\;\;\;\;\;\;\;\;\;\;\;\;\;\;\;\;\;\;\;\;\;\;\;\;\;\;\;\;\;\;\;\left.-P_{w}\left(P_{d}P_{on}+P_{fa}P_{off}\right)\right\} \end{array}$$

## 5. Connection and Secrecy Outage Probabilities

The main objective of the EH-UAV integrated CR system is to use the primary spectrum for its opportunistic transmissions [24,25,26]. Under such an objective, we consider the connection, and secrecy outage probabilities as the performance metrics for reliable and secure communications, respectively. The connection outage probability measures the reliability of the communication link between EH-UAV and SR without E. Similarly, the secrecy outage probability is considered as a performance measure of secure communication between EH-UAV and SR in the presence of E.

### 5.1. Connection Outage Probability

The connection outage probability is defined as the probability that the spectral efficiency (channel capacity) of the UAV-SR link ($C_{CR}$) falls below the target transmission rate ($R_{S1}$) [27]. Thus, the connection outage probability is expressed as

(19) $$P_{S.Out}=Pr\left(C_{CR}<R_{S1}\right).$$

Here, $C_{CR}$ is expressed as

(20) $$C_{CR}=\frac{\left(2\pi -\theta \right)}{2\pi }P_{off}\left(1-P_{fa}\right)\log_{2}\left(1+\gamma_{CR}\right),$$

where $\gamma_{CR}$ is the SNR at the SR, and is defined as $\gamma_{CR}=\frac{|h_{l}{|}^{2}}{\sigma_{sr}^{2}}P_{tx}$. Here, $h_{l}$ denotes the channel coefficient of the secondary link between the UAV and SR, and $\sigma_{sr}^{2}$ is the noise variances at the SR. Similar to $|h_{p}{|}^{2}$, the channel power gain $|h_{l}{|}^{2}$ follows an exponential distribution with a mean of $\omega_{l}$, i.e., $|h_{l}{|}^{2}∼Exp\left(\frac{1}{\omega_{l}}\right)$. Equivalently, the connection outage probability in term of $\gamma_{CR}$ is rewritten as

(21) $$P_{S.Out}=Pr\left(\gamma_{CR}<2^{\left(\frac{R_{S1}2\pi }{P_{off}\left(1-P_{fa}\right)\left(2\pi -\theta \right)}\right)}-1\right).$$

For the evaluation of connection outage probability, the distribution of $\gamma_{CR}$ is needed. Considering the exponential distribution, $|h_{l}{|}^{2}∼Exp\left(\frac{1}{\omega_{l}}\right)$, the connection outage probability is simplified as

(22) $$P_{S.Out}=1-exp\left\{-\frac{\sigma_{sr}^{2}\left(2^{\left(\frac{R_{S1}2\pi }{P_{off}\left(1-P_{fa}\right)\left(2\pi -\theta \right)}\right)}-1\right)}{P_{tx}\omega_{l}}\right\}.$$

### 5.2. Secrecy Outage Probability

As a performance measure of PLS, the secrecy capacity ($C_{s}$) defined as the difference between the capacity of UAV-SR link ($C_{CR}$) and that of the UAV-E link ($C_{e}$) is given as [28],

(23) $$C_{s}={\left[C_{CR}-C_{e}\right]}^{+},$$

where ${\left[x\right]}^{+}=\max\left(x,0\right)$ denotes the larger value between *x* and 0. Moreover, $C_{CR}$ is given in Equation (20) and $C_{e}$ is defined as

(24) $$C_{e}=\frac{\left(2\pi -\theta \right)}{2\pi }P_{off}\left(1-P_{fa}\right)\log_{2}\left(1+\frac{|h_{e}{|}^{2}}{\sigma_{e}^{2}}P_{tx}\right),$$

where $h_{e}$ denotes the channel coefficient of the eavesdropping link between the UAV and E, and $\sigma_{e}^{2}$ is the noise variances at E. The channel power gain $|h_{e}{|}^{2}$ follows an exponential distribution with mean $\omega_{e}$.

The secrecy outage probability is defined as the probability that the $C_{s}$ falls below a target secrecy rate ($R_{S2}$), and is expressed as

(25) $$\begin{array}{cc} P_{Sec.Out} & =Pr\left(C_{s}<R_{S2}\right)\\ \; & =Pr\left\{\frac{1+\frac{|h_{l}{|}^{2}}{\sigma_{sr}^{2}}P_{tx}}{1+\frac{|h_{e}{|}^{2}}{\sigma_{e}^{2}}P_{tx}}<2^{\left(\frac{R_{S2}2\pi }{P_{off}\left(1-P_{fa}\right)\left(2\pi -\theta \right)}\right)}\right\}. \end{array}$$

Equation (25) can be rewritten as

(26) $$\begin{array}{cc} P_{Sec.Out} & =Pr\left\{|h_{l}{|}^{2}<\frac{\sigma_{sr}^{2}\left(2^{\left(\frac{R_{S2}2\pi }{P_{off}\left(1-P_{fa}\right)\left(2\pi -\theta \right)}\right)}-1\right)}{P_{tx}}+{\left|h_{e}\right|}^{2}\frac{\sigma_{sr}^{2}2^{\left(\frac{R_{S2}2\pi }{P_{off}\left(1-P_{fa}\right)\left(2\pi -\theta \right)}\right)}}{\sigma_{e}^{2}}\right\}. \end{array}$$

To evaluate the secrecy outage probability, the distribution of SNRs for the UAV-SR and UAV-E links are required. Considering the distributions $|h_{l}{|}^{2}∼Exp\left(\frac{1}{\omega_{l}}\right)$ and $|h_{e}{|}^{2}∼Exp\left(\frac{1}{\omega_{e}}\right)$, the connection outage probability is obtained as

(27) $$\begin{array}{cc} P_{Sec.Out}= & \int_{0}^{\infty }\left\{1-exp\left(-\frac{\sigma_{sr}^{2}\left(2^{\left(\frac{R_{S2}2\pi }{P_{off}\left(1-P_{fa}\right)\left(2\pi -\theta \right)}\right)}-1\right)}{\omega_{l}P_{tx}}-\frac{\sigma_{sr}^{2}2^{\left(\frac{R_{S2}2\pi }{P_{off}\left(1-P_{fa}\right)\left(2\pi -\theta \right)}\right)}}{\omega_{l}\sigma_{e}^{2}}z\right)\right\}\\ \; & \times \frac{1}{\omega_{e}}exp\left(-\frac{z}{\omega_{e}}\right)dz. \end{array}$$

By simplifying Equation (27), the secrecy outage probability is expressed as

(28) $$\begin{array}{c} P_{Sec.Out}=1-\left(\frac{\sigma_{e}^{2}\omega_{l}}{\sigma_{e}^{2}\omega_{l}+2^{\left(\frac{R_{S2}2\pi }{P_{off}\left(1-P_{fa}\right)\left(2\pi -\theta \right)}\right)}\sigma_{sr}^{2}\omega_{e}}\right)exp\left\{-\frac{\sigma_{sr}^{2}\left(2^{\left(\frac{R_{S2}2\pi }{P_{off}\left(1-P_{fa}\right)\left(2\pi -\theta \right)}\right)}-1\right)}{\omega_{l}P_{tx}}\right\}. \end{array}$$

## 6. Maximization of Total Residual Energy

The total residual energy and two outage probabilities, including imperfect sensing, i.e., false alarm and detection probabilities, are too complicated to formulate and solve as an optimization problem, i.e., to maximize the total residual energy under outage constraints. For making the optimization problem tractable, the perfect spectrum sensing is assumed, i.e., $P_{d}=1$ and $P_{fa}=\;0$. Under this assumption, the approximated total residual energy (${\tilde{E}}_{R,Tot}$), the approximated connection and secrecy outage probabilities (${\tilde{P}}_{S.Out}$), (${\tilde{P}}_{Sec.Out}$), respectively, are simplified. With these approximated expressions, we consider the relaxed (approximated) optimization problems. To make the approximation reasonable, a high primary signal power is assumed, i.e., $|h_{p}{|}^{2}P_{x}\gg \sigma^{2}$.

Two maximization problems of ${\tilde{E}}_{R,Tot}$ are considered under two separate constraints: the connection outage probability constraint $\left({\tilde{P}}_{S.Out}\leq \varphi_{1}\right)$, and secrecy outage probability constraint $\left({\tilde{P}}_{Sec.Out}\leq \varphi_{2}\right)$. $\varphi_{1}$ and $\varphi_{2}$ denote the connection and secrecy outage constraint thresholds, respectively.

### 6.1. Residual Energy Maximization under Connection Outage Constraint

In this subsection, a connection outage constraint is considered for a scenario without E. The optimization problem to maximize the residual energy with a connection outage constraint is formulated as

(29) $$\begin{array}{cc} \max_{\theta ,P_{tx}} & \;\;{\tilde{E}}_{R,Tot}\left(\theta ,P_{tx}\right)\\ s.t. & \;\;{\tilde{P}}_{S.Out}\left(\theta ,P_{tx}\right)\leq \varphi_{1}. \end{array}$$

The optimization problem includes two variables: the length of the sensing phase (θ) and the transmit power ($P_{tx}$). To find the optimal pair, $\theta^{o}$ and $P_{tx}^{o}$, i.e., the solution to Equation (29), we consider two subproblems with each variable while fixing the other variable.

First, the optimization problem with respect to θ with a fixed $P_{tx}$ is considered as follows:

(30) $$\begin{array}{cc} \max_{\theta } & \;\;\frac{r}{v}\theta \left\{\left\{\left(1-\rho \right)\left(\eta P_{on}{\left|h_{p}\right|}^{2}P_{x}-P_{w}\right)\right\}-P_{\delta }\right\}+\frac{r}{v}\left(2\pi -\theta \right)\left\{\eta P_{on}{\left|h_{p}\right|}^{2}P_{x}-P_{on}P_{w}-P_{off}P_{tx}\right\}\\ s.t. & \;\;\;\;\;1-exp\left\{-\frac{\sigma_{sr}^{2}\left(2^{\left(\frac{R_{S1}2\pi }{P_{off}\left(2\pi -\theta \right)}\right)}-1\right)}{P_{tx}\omega_{l}}\right\}\leq \varphi_{1}. \end{array}$$

For finding the solution, the characteristics of the objective and constraint functions, i.e., the approximated residual energy and connection outage probability, with respect to θ are investigated.

**Lemma** **1.** *For a fixed $P_{tx}$, ${\tilde{E}}_{R,Tot}\left(\theta \right)$, and ${\tilde{P}}_{S.Out}\left(\theta \right)$ are monotonically increasing functions of θ.*

**Proof.**  Under a general system configuration, the power harvested in the sensing phase $\left\{\left(1-\rho \right)\left(\eta P_{on}{\left|h_{p}\right|}^{2}P_{x}-P_{w}\right)-P_{\delta }\right\}$ is larger than the energy harvested in the transmission phase $\left(\eta P_{on}{\left|h_{p}\right|}^{2}P_{x}-P_{on}P_{w}-P_{off}P_{tx}\right)$. Otherwise, the optimal approach to maximize the residual energy is θ=0, which becomes a trivial solution. Therefore, ${\tilde{E}}_{R,Tot}\left(\theta \right)$ is a linearly increasing function of θ. The capacity of the secondary link $C_{CR}$ linearly decreases with θ under the perfect sensing assumption because the transmission duration linearly decreases with θ. Hence, the connection outage probability is also a monotonically increasing function of θ. □

Based on Lemma 1, we can find the solution to Equation (30) as follows:

**Lemma** **2.** *For a fixed $P_{tx}$, the θ maximizing the total residual energy under a connection outage constraint, i.e., the solution to Equation (30), is given as*

(31) $$\theta^{o}=2\pi -\frac{R_{S1}2\pi }{P_{off}}{\left\{\log_{2}\left(1-\frac{P_{tx}\omega_{l}\ln\left({\bar{\varphi }}_{1}\right)}{\sigma_{sr}^{2}}\right)\right\}}^{-1}$$

*where ${\bar{\varphi }}_{1}=1-\varphi_{1}$.*

**Proof.**  The objective function ${\tilde{E}}_{R,Tot}$ monotonically increases with θ, therefore the optimal policy is to increase θ while satisfying the constraint. Additionally, the connection outage probability is also an increasing function of θ. Therefore, the θ at which the connection outage probability approaches its upper bound is considered. Hence, the solution to Equation (30) is $\theta^{o}$ such that ${\tilde{P}}_{S.Out}\left(\theta^{o}\right)=\varphi_{1}$. □

Next, the optimization problem with respect to $P_{tx}$ with a fixed θ is formulated as

(32) $$\begin{array}{cc} \max_{P_{tx}} & \;\;\frac{r}{v}\theta \left\{\left\{\left(1-\rho \right)\left(\eta P_{on}{\left|h_{p}\right|}^{2}P_{x}-P_{w}\right)\right\}-P_{\delta }\right\}+\frac{r}{v}\left(2\pi -\theta \right)\left\{\eta P_{on}{\left|h_{p}\right|}^{2}P_{x}-P_{on}P_{w}-P_{off}P_{tx}\right\}\\ s.t. & \;\;\;\;\;1-exp\left\{-\frac{\sigma_{sr}^{2}\left(2^{\left(\frac{R_{S1}2\pi }{P_{off}\left(2\pi -\theta \right)}\right)}-1\right)}{P_{tx}\omega_{l}}\right\}\leq \varphi_{1}. \end{array}$$

By investigating the objective and constraint functions with respect to $P_{tx}$, we can find the following property and the solution to Equation (32).

**Lemma** **3.** *For a given θ, ${\tilde{E}}_{R,Tot}\left(P_{tx}\right)$ and ${\tilde{P}}_{S.Out}\left(P_{tx}\right)$ are monotonically decreasing functions of $P_{tx}$*

**Proof.**  The secondary transmission power $P_{tx}$ is the consumed power in the transmission phase. Therefore, it can be shown that ${\tilde{E}}_{R,Tot}\left(P_{tx}\right)$ monotonically decreases with $P_{tx}$. Form the formula for connection outage probability, it is also shown that ${\tilde{P}}_{S.Out}\left(P_{tx}\right)$ is a monotonically decreasing function of $P_{tx}$. □

Based on Lemma 3, we can find the solution to Equation (32) as follows:

**Lemma** **4.** *For a fixed θ, the $P_{tx}$ maximizing the total residual energy with a constraint of the connection outage probability is given by*

(33) $$P_{tx}^{o}=\frac{\sigma_{sr}^{2}}{\omega_{l}\ln\left({\bar{\varphi }}_{1}\right)}\left\{1-2^{\left(\frac{R_{S1}2\pi }{P_{off}\left(2\pi -\theta \right)}\right)}\right\}$$

**Proof.**  With the same approach in the proof of Lemma 2, it can easily be shown that the solution to Equation (32) is $P_{tx}^{o}$ such that ${\tilde{P}}_{S.Out}\left(P_{tx}^{o}\right)=\varphi_{1}$. □

In the procedure to find the solutions to both subproblems, it is shown that both solutions are obtained when the inequality constraint is satisfied with equality. Thus, we can conclude that the joint optimal solution satisfies the outage constraint at the boundary, i.e., ${\tilde{P}}_{S.Out}=\varphi_{1}$. Based on this observation, the original joint optimization problem with two variables is converted into two optimization problems with a single variable with respect to θ and $P_{tx}$. The first problem is given as

(34) $$\begin{array}{cc} \max_{P_{tx}} & \;\;{\tilde{E}}_{R,Tot}\left(\theta^{o},P_{tx}\right) \end{array}$$

where ${\tilde{E}}_{R,Tot}\left(\theta^{o},P_{tx}\right)$ is defined by substituting ${\tilde{E}}_{R,Tot}\left(\theta ,P_{tx}\right)$ with $\theta^{o}$ in Lemma 2. The other problem is expressed as

(35) $$\begin{array}{cc} \max_{\theta } & \;\;{\tilde{E}}_{R,Tot}\left(\theta ,P_{tx}^{o}\right) \end{array}$$

where ${\tilde{E}}_{R,Tot}\left(\theta ,P_{tx}^{o}\right)$ is defined by substituting ${\tilde{E}}_{R,Tot}\left(\theta ,P_{tx}\right)$ with $P_{tx}^{o}$ in Lemma 4. The objective functions of both Equations (34) and (35), ${\tilde{E}}_{R,Tot}\left(\theta^{o},P_{tx}\right)$ and ${\tilde{E}}_{R,Tot}\left(\theta ,P_{tx}^{o}\right)$, are the concave functions of $P_{tx}$ and θ, respectively. The concavity of both functions is shown in a numerical way. Based on the concavity of the objective functions, we can solve one of the problems of Equations (34) and (35) using the first-order optimality conditions $\frac{d{\tilde{E}}_{R,Tot}\left(\theta^{o},P_{tx}\right)}{dP_{tx}}=0$ and $\frac{d{\tilde{E}}_{R,Tot}\left(\theta ,P_{tx}^{o}\right)}{d\theta }=0$, respectively. However, the closed-form expression is hard to obtain. As an alternative, we can solve the standard convex optimization problem using a numerical method, e.g., gradient descent and steepest descent algorithms. After finding $P_{tx}^{o}$ (or $\theta^{o}$) with a numerical method, the remaining solution $\theta^{o}$ (or $P_{tx}^{o}$) can be obtained using the equality constraint, i.e., ${\tilde{P}}_{S,Out}\left(\theta^{o},P_{tx}^{o}\right)=\varphi_{1}$.

### 6.2. Residual Energy Maximization under Secrecy Outage Constraint

We now consider a scenario with E. To avoid eavesdropping, a secrecy outage probability with a given threshold $\varphi_{2}$ is considered as a constraint. The optimization problem to maximize the residual energy with a secrecy outage constraint is formulated as

(36) $$\begin{array}{cc} \max_{\theta ,P_{tx}} & \;\;{\tilde{E}}_{R,Tot}\left(\theta ,P_{tx}\right)\\ s.t. & \;\;{\tilde{P}}_{Sec.Out}\left(\theta ,P_{tx}\right)\leq \varphi_{2}. \end{array}$$

To find a jointly optimal solution to Equation (36), i.e., $\theta^{*}$ and $P_{tx}^{*}$, we consider two subproblems as in the previous subsection. The first subproblem, i.e., the optimization problem with respect to θ with a fixed $P_{tx}$, is as follows:

(37) $$\begin{array}{cc} \max_{\theta } & \;\;\frac{r}{v}\theta \left\{\left\{\left(1-\rho \right)\left(\eta P_{on}{\left|h_{p}\right|}^{2}P_{x}-P_{w}\right)\right\}-P_{\delta }\right\}+\frac{r}{v}\left(2\pi -\theta \right)\left\{\eta P_{on}{\left|h_{p}\right|}^{2}P_{x}-P_{on}P_{w}-P_{off}P_{tx}\right\}\\ s.t. & \;\;1-\left(\frac{\sigma_{e}^{2}\omega_{l}}{\sigma_{e}^{2}\omega_{l}+2^{\left(\frac{R_{S2}2\pi }{P_{off}\left(2\pi -\theta \right)}\right)}\sigma_{sr}^{2}\omega_{e}}\right)exp\left\{-\frac{\sigma_{sr}^{2}\left(2^{\left(\frac{R_{S2}2\pi }{P_{off}\left(2\pi -\theta \right)}\right)}-1\right)}{\omega_{l}P_{tx}}\right\}\leq \varphi_{2}. \end{array}$$

In addition to Lemma 1, it can be readily shown that the approximated secrecy outage probability ${\tilde{P}}_{Sec,Out}\left(\theta \right)$ with a fixed $P_{tx}$ is a monotone increasing function of θ. Similar to Lemma 2, the solution to Equation (37) can be obtained

**Lemma** **5.** For a fixed $P_{tx}$, the optimal solution to Equation (37) is given by $\theta^{*}$ such that ${\tilde{P}}_{Sec,Out}\left(\theta^{*}\right)=\varphi_{2}$. Especially, when SNR of the legitimate link is much higher than that of the eavesdropping link, i.e., $\frac{\omega_{l}}{\sigma_{sr}^{2}}\gg \frac{\omega_{e}}{\sigma_{e}^{2}}$, the optimal $\theta^{*}$ can be approximated given by

(38) $$\begin{array}{c} \theta^{*}\approx 2\pi -\frac{R_{S2}2\pi }{P_{off}}{\left\{\log_{2}\left(\frac{\omega_{l}\ln\left({\bar{\varphi }}_{2}\right)+\frac{\sigma_{sr}^{2}}{P_{tx}}}{\frac{\sigma_{sr}^{2}}{P_{tx}}+\frac{\sigma_{sr}^{2}\omega_{e}}{\sigma_{e}^{2}}}\right)\right\}}^{-1} \end{array}$$

*where ${\bar{\varphi }}_{2}=1-\varphi_{2}$.*

**Proof.**  Same as in the proof of Lemma 2, it can be easily shown that the solution to Equation (37) is $\theta^{*}$ such that ${\tilde{P}}_{Sec.Out}\left(\theta^{*}\right)=\varphi_{2}$. Additionally, we have

(39) $$\begin{array}{cc} {\tilde{P}}_{Sec.Out}\left(\theta^{*}\right) & =\varphi_{2}\\ {\bar{\varphi }}_{2} & ={\left(1+\frac{2^{\left(\frac{R_{S2}2\pi }{P_{off}\left(2\pi -\theta^{*}\right)}\right)}\sigma_{sr}^{2}\omega_{e}}{\sigma_{e}^{2}\omega_{l}}\right)}^{-1}exp\left\{-\frac{\sigma_{sr}^{2}\left(2^{\left(\frac{R_{S2}2\pi }{P_{off}\left(2\pi -\theta^{*}\right)}\right)}-1\right)}{\omega_{l}P_{tx}}\right\}\\ \ln\left({\bar{\varphi }}_{2}\right) & =-\ln\left(1+\frac{2^{\left(\frac{R_{S2}2\pi }{P_{off}\left(2\pi -\theta^{*}\right)}\right)}\sigma_{sr}^{2}\omega_{e}}{\sigma_{e}^{2}\omega_{l}}\right)-\frac{\sigma_{sr}^{2}\left(2^{\left(\frac{R_{S2}2\pi }{P_{off}\left(2\pi -\theta^{*}\right)}\right)}-1\right)}{\omega_{l}P_{tx}}\\ \ln\left({\bar{\varphi }}_{2}\right) & \approx -\frac{2^{\left(\frac{R_{S2}2\pi }{P_{off}\left(2\pi -\theta^{*}\right)}\right)}\sigma_{sr}^{2}\omega_{e}}{\sigma_{e}^{2}\omega_{l}}-\frac{\sigma_{sr}^{2}\left(2^{\left(\frac{R_{S2}2\pi }{P_{off}\left(2\pi -\theta^{*}\right)}\right)}-1\right)}{\omega_{l}P_{tx}}. \end{array}$$

In Equation (39), we use that ln(1+x)≈x when x≪1. By rearranging the last equation with respect to $\theta^{*}$, we can complete the proof. □

The second subproblem, i.e., the optimization problem with respect to $P_{tx}$ with a fixed θ is formulated as

(40) $$\begin{array}{cc} \max_{P_{tx}} & \;\;\frac{r}{v}\theta \left\{\left\{\left(1-\rho \right)\left(\eta P_{on}{\left|h_{p}\right|}^{2}P_{x}-P_{w}\right)\right\}-P_{\delta }\right\}+\frac{r}{v}\left(2\pi -\theta \right)\left\{\eta P_{on}{\left|h_{p}\right|}^{2}P_{x}-P_{on}P_{w}-P_{off}P_{tx}\right\}\\ s.t. & \;\;1-\left(\frac{\sigma_{e}^{2}\omega_{l}}{\sigma_{e}^{2}\omega_{l}+2^{\left(\frac{R_{S2}2\pi }{P_{off}\left(2\pi -\theta \right)}\right)}\sigma_{sr}^{2}\omega_{e}}\right)exp\left\{-\frac{\sigma_{sr}^{2}\left(2^{\left(\frac{R_{S2}2\pi }{P_{off}\left(2\pi -\theta \right)}\right)}-1\right)}{\omega_{l}P_{tx}}\right\}\leq \varphi_{2}. \end{array}$$

The objective and constraint functions in Equation (40) are both monotonically decreasing functions of $P_{tx}$. Thus, the solution to Equation (40) can be obtained as follows:

**Lemma** **6.** *For a fixed θ, the solution to Equation (40) is given by*

(41) $$\begin{array}{c} P_{tx}^{*}=\frac{\sigma_{sr}^{2}}{\omega_{l}}\left(2^{\left(\frac{R_{S2}2\pi }{P_{off}\left(2\pi -\theta \right)}\right)}-1\right){\left\{\ln\left(\frac{\sigma_{e}^{2}\omega_{l}}{2^{\left(\frac{R_{S2}2\pi }{P_{off}\left(2\pi -\theta \right)}\right)}{\bar{\varphi }}_{2}\sigma_{sr}^{2}\omega_{e}+{\bar{\varphi }}_{2}\sigma_{e}^{2}\omega_{l}}\right)\right\}}^{-1} \end{array}$$

**Proof.**  With the same approach to the proof of Lemma 4, it can be easily shown that the solution to Equation (40) is $P_{tx}^{*}$ such that ${\tilde{P}}_{Sec.Out}\left(P_{tx}^{*}\right)=\varphi_{2}$. Therefore, we can rewrite as follow:

(42) $$\begin{array}{cc} {\tilde{P}}_{Sec.Out}\left(P_{tx}^{*}\right)= & \varphi_{2}\\ 1-\varphi_{2}= & \left(\frac{\sigma_{e}^{2}\omega_{l}}{2^{\left(\frac{R_{S2}2\pi }{P_{off}\left(2\pi -\theta \right)}\right)}\sigma_{sr}^{2}\omega_{e}+\sigma_{e}^{2}\omega_{l}}\right)exp\left\{-\frac{\sigma_{sr}^{2}\left(2^{\left(\frac{R_{S2}2\pi }{P_{off}\left(2\pi -\theta \right)}\right)}-1\right)}{\omega_{l}P_{tx}^{*}}\right\}\\ \ln\left({\bar{\varphi }}_{2}\right)= & \ln\left(\frac{\sigma_{e}^{2}\omega_{l}}{2^{\left(\frac{R_{S2}2\pi }{P_{off}\left(2\pi -\theta \right)}\right)}\sigma_{sr}^{2}\omega_{e}+\sigma_{e}^{2}\omega_{l}}\right)-\frac{\sigma_{sr}^{2}\left(2^{\left(\frac{R_{S2}2\pi }{P_{off}\left(2\pi -\theta \right)}\right)}-1\right)}{\omega_{l}P_{tx}^{*}} \end{array}$$

Rearranging Equation (42), we can have Equation (41) □

From Lemmas 5 and 6, it can be seen that the joint optimal solution holds ${\tilde{P}}_{Sec.Out}=\varphi_{2}$. Thus, the original joint optimization problem with two variables is converted into two optimization problems as follows:

(43) $$\begin{array}{cc} \max_{P_{tx}} & \;\;{\tilde{E}}_{R,Tot}\left(\theta^{*},P_{tx}\right) \end{array}$$

where ${\tilde{E}}_{R,Tot}\left(\theta^{*},P_{tx}\right)$ denotes ${\tilde{E}}_{R,Tot}\left(\theta ,P_{tx}\right)$ with $\theta =\theta^{*}$ in Lemma 5, and

(44) $$\begin{array}{cc} \max_{\theta } & \;\;{\tilde{E}}_{R,Tot}\left(\theta ,P_{tx}^{*}\right) \end{array}$$

where ${\tilde{E}}_{R,Tot}\left(\theta ,P_{tx}^{*}\right)$ is defined by substituting ${\tilde{E}}_{R,Tot}\left(\theta ,P_{tx}\right)$ with $P_{tx}^{*}$ in Lemma 6.

Here, we can numerically verify that ${\tilde{E}}_{R,Tot}\left(\theta^{*},P_{tx}\right)$ and ${\tilde{E}}_{R,Tot}\left(\theta ,P_{tx}^{*}\right)$ are the concave functions of $P_{tx}$ and θ, respectively. Therefore, we can numerically find the final solution with a same way in the previous subsection.

## 7. Numerical Results and Discussion

In this section, we perform the numerical simulations to verify the analytical results and provide a discussion of the results. We summarize the default system configuration (unless otherwise stated) in Table 1. The values of parameters are set to validate the behavior of system. The exact functions with lower values of sensing error, i.e., $P_{d}=0.85$, are considered to compute the performance evaluation in term of the residual energy.

**Figure 2:**
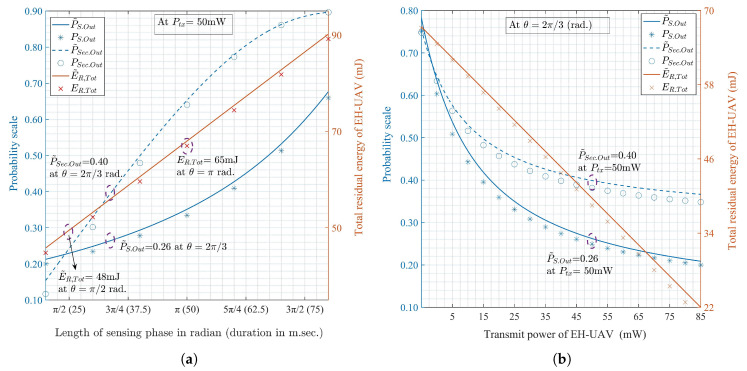
Total residual energy, connection and secrecy outage probabilities, and their approximation under perfect sensing with respect to (**a**) length of sensing phase (duration) with a fixed transmit power, (**b**) transmit power for a fixed length of sensing phase.

Figure 2a validates that ${\tilde{E}}_{R,Tot}$, ${\tilde{P}}_{S.Out}$, and ${\tilde{P}}_{Sec.Out}$ are the monotonically increasing functions with respect to θ for a fixed $P_{tx}$. The maximum of ${\tilde{E}}_{R,Tot}$ is achieved at the upper bound of θ, whereas the lower-bound provides the maximum of ${\tilde{P}}_{S.Out}$ and ${\tilde{P}}_{Sec.Out}$. Thus, the optimal policy to maximize the total residual energy is to increase θ and decrease $P_{tx}$. In contrast, Figure 2b verifies that ${\tilde{E}}_{R,Tot}$, ${\tilde{P}}_{S.Out}$, and ${\tilde{P}}_{Sec.Out}$ are the monotonically decreasing functions with respect to $P_{tx}$ for a given θ. The maximum ${\tilde{E}}_{R,Tot}$ is obtained with the lower-bound of $P_{tx}$, whereas the upper-bound provides the maximum of ${\tilde{P}}_{S.Out}$ and ${\tilde{P}}_{Sec.Out}$. Here, decreasing θ and increasing $P_{tx}$ is optimal from the perspective of connection and secrecy outage performances without the consideration of residual energy. These results exhibit the inherent trade-off between the residual energy and transmission performances for an EH-UAV.

**Figure 3:**
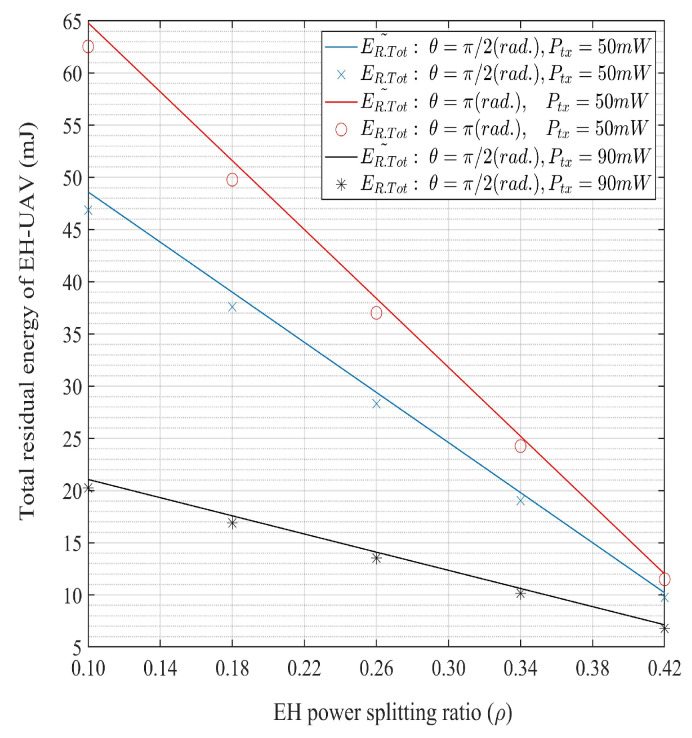
Total residual energy and its approximation with respect to energy harvesting (EH) power splitting ratio for θ={π/2,π} and $P_{tx}=\left\{50,90\right\}$ mW.

**Figure 4:**
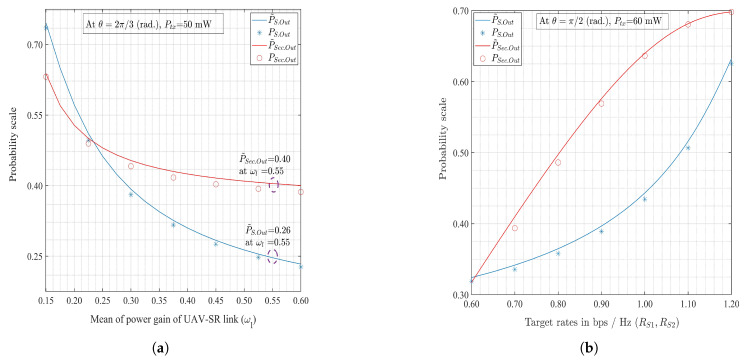
Monotonic functions of connection outage probability, secrecy outage probability vs. (**a**) mean (expectation) of $|h_{l}{|}^{2}$ and (**b**) target rates.

Figure 3 shows the variations of ${\tilde{E}}_{R,Tot}$ for the EH splitting ratio (ρ) depending on θ={π/2,π} and $P_{tx}=\left\{50,90\right\}$ mW. It is seen that ${\tilde{E}}_{R,Tot}$ is a decreasing function with respect to ρ. A higher ${\tilde{E}}_{R,Tot}$ is achieved with the lower values of ρ because (1−ρ)-fraction of the received power is used for the EH process. Figure 4a shows the connection and secrecy outage probabilities, ${\tilde{P}}_{S.Out}$ and ${\tilde{P}}_{Sec.Out}$, respectively, as the functions of the expected channel gain, $\omega_{l}$. The secondary channel and secrecy capacities, $C_{CR}$, and $C_{s}$, respectively, increase with $\omega_{l}$. Thus, both ${\tilde{P}}_{S.Out}$, and ${\tilde{P}}_{Sec.Out}$ decrease with the increase in $\omega_{l}$. Figure 4b shows that both the connection and secrecy outage probabilities increase with the target transmission rate ($R_{S1}$) and target secrecy rate ($R_{S2}$), respectively.

**Figure 5:**
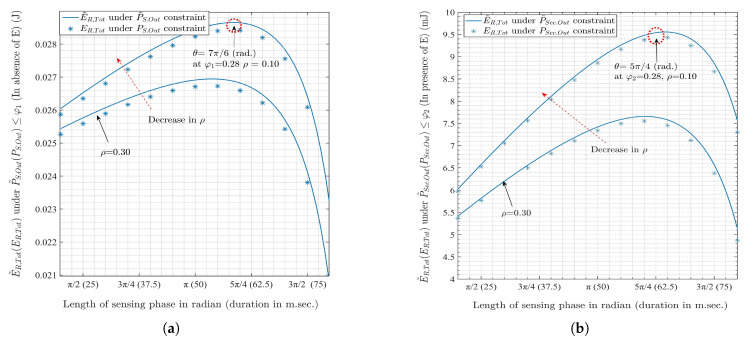
Exact and approximated total residual energy as a function of θ for EH-unmanned aerial vehicle (UAV) under (**a**) connection outage constraint, (**b**) secrecy outage constraint.

**Figure 6:**
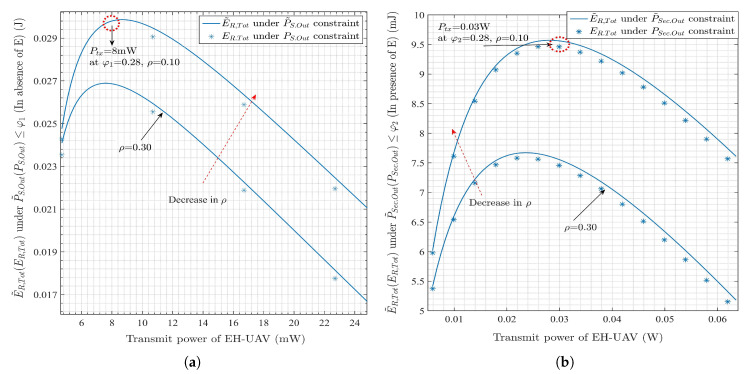
Exact and approximated total residual energy as a function of $P_{tx}$ for EH-UAV under (**a**) connection outage constraint, (**b**) secrecy outage constraint.

In addition, from Figure 2, Figure 3, Figure 4, Figure 5 and Figure 6, it can be seen that the approximated ${\tilde{E}}_{R,Tot}$, ${\tilde{P}}_{S.Out}$, and ${\tilde{P}}_{Sec.Out}$ with the perfect channel sensing are sufficiently accurate to be used in the proposed optimization problems instead of the exact values.

Figure 5a,b depict the total residual energies and their approximations under ${\tilde{P}}_{S.Out}\leq \varphi_{1}$ and ${\tilde{P}}_{Sec.Out}\leq \varphi_{2}$, respectively, over θ. Similarly, Figure 6a,b show the exact and approximated residual energies with the constraints of connection and secrecy outage probabilities, respectively, over $P_{tx}$. For Figure 5 and Figure 6, $R_{S1}$ and $R_{S2}$ are set at 0.25 bps/Hz, and 0.55 bps/Hz, respectively. Moreover, $\varphi_{1}$ and $\varphi_{2}$ are set at 0.28. In Figure 5a,b, $P_{tx}$ associated with θ is determined optimally using Lemmas 2 and 5, respectively. Under the considered scenarios, a higher θ results in the higher $P_{tx}$ because a large θ during the flight leads to a decrease in the length of transmission phase (2π−θ), which results in higher $P_{tx}$ requirements to meet the target rates, $R_{S1}$ and $R_{S2}$. Thus, it can be inferred that initially, with a large θ, ${\tilde{E}}_{R,Tot}$ increases since the EH-UAV gets more opportunities to harvest the energy during its flight. After reaching the maximum point, ${\tilde{E}}_{R,Tot}$ goes on decreasing with $P_{tx}$ to meet the target rate. A similar phenomenon is observed in Figure 6a,b, with the higher $P_{tx}$ resulting in higher ${\tilde{E}}_{R,Tot}$ initially. The reason is that the increment in $P_{tx}$ also increases θ, which is proportional to the opportunities available for EH-UAV to harvest the energy. After achieving the maximum point, ${\tilde{E}}_{R,Tot}$ decreases with the $P_{tx}$, since any further increase leads to only a small increment in θ. From the Figure 5,b, it is observed that the maximum value of θ, i.e., 7π/6 rad. and 5π/4 rad., in the absence and presence of E, respectively, which validate the numerically obtained solutions to Equations (35) and (44), respectively. In Figure 6a,b, the maximum value of $P_{tx}$ is 8 mW, and 0.03 W, in the absence and presence of E, respectively, and validate the numerically obtained solutions to Equations (34) and (43), respectively. One of the important observations in Figure 5 and Figure 6 is that the four different residual energy functions are the concave functions with respect to θ or $P_{tx}$ when the other variable is optimally determined by the equality constraints.

**Figure 7:**
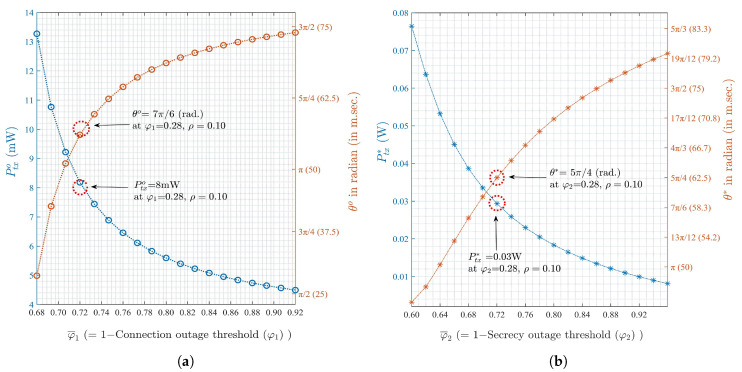
Optimal lengths of sensing phase (duration) and optimal transmit powers for EH-UAV over (**a**) connection outage threshold ($\varphi_{1}$), (**b**) secrecy outage threshold ($\varphi_{2}$).

Finally, Figure 7a,b show the numerically obtained joint optimal solution pairs to the optimization problems in Equations (29) and (36), i.e., ($\theta^{o}$, $P_{tx}^{o}$) and ($\theta^{*}$, $P_{tx}^{*}$), respectively. It can be observed that the $P_{tx}^{*}>P_{tx}^{o}$ and $\theta^{*}>\theta^{o}$, which validates the fact that the EH-UAV must increase its $P_{tx}$ to meet $R_{S2}$ in the presence of E. This, in turn, reduces the required length of transmit phase (2π−θ) for EH-UAV, and consequently the value of θ also increases. Thus, the requirements for the optimal $P_{tx}$ and θ also increase. Our results identify the requirements of $P_{tx}$ and θ for the maximization of residual energy while maintaining reliable and secure communications (in the presence and absence of E, respectively), and provide the guidelines in designing an energy-harvesting UAV-based CR system.

## 8. Conclusions

Cognitive radio (CR) is a promising enabler communication technology to mitigate the spectrum scarcity and under-utilization issues in futuristic networks such as the IoT and 5G. The integration of UAVs in CR systems enhances the sensing performance. The wireless energy harvesting technique effectively alleviates the energy scarcity in the UAV-enabled wireless networks. Furthermore, most of the UAV based scenarios demand reliable and secure communications. In this paper, we considered that the UAV, with the limited energy-budget and a circular flight track (around a ground-mounted primary transmitter), harvests RF energy from the primary transmissions and uses the primary spectrum opportunistically. The closed-form analytical expressions for the residual energy, connection, and secrecy outage probabilities were derived to investigate the performances of energy-harvesting, reliable, and secure communications, in the absence and presence of an eavesdropper, respectively. The optimization problems were constructed by exploiting the trade-off between monotonic approximated functions, and the analytical solutions, i.e., the optimal lengths of sensing phase and transmit powers, are identified under two different scenarios for the UAV. The numerical simulations verified the proposed theoretical analysis, and demonstrated the impact of system parameters on the residual energy performance while ensuring the reliable and secure communication for UAV.

## Figures and Tables

**Table 1:** System configuration for numerical simulations.

Symbol	Description
$P_{off},P_{on}$	0.50 ( fair model of channel occupancy)
$P_{x}$	1.05 W
$P_{d}$	0.85 (interference probability to PT is 15% or less for the imperfect sensing)
$\omega_{p}$	0.95
$\omega_{e}$	0.82
$\omega_{l}$	0.55
η	0.80
ρ	0.10
*h*	100m
$\sigma^{2}$	0.10 W
$\sigma_{sr}^{2}$	0.78 W
$\sigma_{e}^{2}$	0.22 W
$P_{w},P_{\delta }$	0.50 mW
$f_{s}$	50 kHz
$R_{S1}$	0.30 bps/Hz
$R_{S2}$	0.60 bps/Hz
